# Junctional Failures Following Long-Level Fusion to L5 in Elderly Patients: Impact of Spinopelvic Alignment and L5–S1 Disc Degeneration

**DOI:** 10.3390/medicina62020411

**Published:** 2026-02-21

**Authors:** In-Seok Son, Yong-Chan Kim, Sung-Min Kim, Xiongjie Li, Maolin Jin, Young-Jik Lee, Seung-Hyun Sim, Kee-Yong Ha

**Affiliations:** Department of Orthopaedic Surgery, College of Medicine, Kyung Hee University, Kyung Hee University Hospital at Gangdong, Seoul 05278, Republic of Korea; wiselove@naver.com (I.-S.S.); yckimspine@gmail.com (Y.-C.K.);

**Keywords:** junctional failure, adult spinal deformity, spinopelvic alignment, lumbosacral disc degeneration

## Abstract

*Background and Objectives*: Long spinal fusion terminating at L5 remains controversial because of the risk of postoperative junctional failure. Although degeneration of the residual L5–S1 disc has been suggested as a contributing factor, the relative impact of disc degeneration versus sagittal spinopelvic alignment on different junctional failure patterns has not been fully clarified. *Materials and Methods*: This retrospective cohort study included 47 patients aged ≥60 years who underwent ≥5-level thoracolumbar fusion ending at L5 with a minimum follow-up of 2 years. Junctional failures were classified as proximal junctional failure (PJF) or distal junctional failure (DJF). Preoperative L5–S1 disc degeneration was assessed using modified Weiner and Pfirrmann classifications. Spinopelvic parameters were measured preoperatively, postoperatively, and at final follow-up. Junctional failure–free survival was analyzed using the Kaplan–Meier method, and risk factors were explored using Cox proportional hazards models. *Results*: Junctional failures occurred in 28 patients (59.6%), including 16 PJFs (34.0%) and 10 DJFs (21.3%). Lower grades of L5–S1 disc degeneration (Weiner grades 0–1) were more frequently associated with PJFs, whereas higher grades (≥2) were predominantly associated with DJFs (*p* = 0.024). Multivariate analysis showed that preoperative thoracolumbar kyphosis (hazard ratio [HR] = 1.164), preoperative T1 pelvic angle (HR = 1.269), and postoperative pelvic incidence–lumbar lordosis mismatch (HR = 0.877) as significant risk factors for PJF. Postoperative proximal junctional angle (HR = 0.899) and lumbar lordosis (HR = 0.920) were independently associated with DJF. *Conclusions*: Sagittal spinopelvic alignment parameters appear to have a greater influence on junctional failure patterns than residual L5–S1 disc degeneration in long fusions terminating at L5. Adequate sagittal correction should be prioritized to reduce the risk of both proximal and distal junctional failures.

## 1. Introduction

Long spinal fusion is a well-established surgical strategy for the correction of adult spinal deformity (ASD); however, the choice of the distal fusion level remains controversial. In particular, whether to terminate a long construct at L5 or extend fixation to the sacrum continues to be debated, especially in elderly patients with degenerative pathology. While fusion to L5 preserves lumbosacral motion and may reduce surgical invasiveness, it also exposes the remaining L5–S1 segment to increased mechanical stress, potentially leading to postoperative sagittal decompensation and junctional failure [1,2,3,4].

Postoperative sagittal decompensation following long fusion can manifest as mechanical failure at different locations, including proximal junctional failure (PJF), distal junctional failure (DJF), or intra-construct complications such as rod fracture and pseudarthrosis [5,6,7]. Although risk factors for proximal junctional kyphosis and failure have been extensively investigated, distal junctional failure after fusion ending at L5 remains less well understood and has been relatively underreported. Systematic reviews have demonstrated a wide range in the incidence of distal junctional kyphosis and failure, largely depending on follow-up duration and patient characteristics [8].

Degeneration of the residual L5–S1 disc has been proposed as a key contributor to distal mechanical complications following long fusion terminating at L5. Previous studies have reported substantial rates of progressive disc degeneration and subsequent reoperation at the lumbosacral junction after long constructs stopping at L5 [1,2,9]. However, the relationship between preoperative disc degeneration and the specific pattern of junctional failure—proximal versus distal—has not been clearly defined. In addition, preserved segmental motion at a relatively healthy L5–S1 disc may paradoxically increase mechanical stress transfer to adjacent junctions after fusion, as motion and mechanical load may be concentrated at the transition zone between the rigid long construct and the remaining mobile lumbosacral segment, potentially influencing failure patterns [2,4]. This paradox arises because a rigid long construct can concentrate motion and mechanical load at the remaining mobile L5–S1 segment, creating a stress transition zone that may increase biomechanical demand at adjacent junctions.

Beyond disc degeneration, sagittal spinopelvic alignment plays a critical role in postoperative mechanical stability following long spinal fusion. Parameters such as pelvic incidence–lumbar lordosis (PI–LL) mismatch, global sagittal balance, and thoracolumbar kyphosis have been consistently reported to influence mechanical complications after long fusion procedures [10,11,12]. However, despite these observations, few studies have simultaneously evaluated the relative contributions of residual L5–S1 disc degeneration and sagittal alignment parameters to both proximal and distal junctional failure patterns in patients undergoing long fusion terminating at L5 [13,14].

Therefore, the purpose of this study was to identify risk factors for junctional failure following long thoracolumbar fusion ending at L5, with particular emphasis on the interaction between preoperative L5–S1 disc degeneration and sagittal spinopelvic alignment. We further sought to clarify whether distinct radiographic and biomechanical profiles are associated with proximal versus distal junctional failure patterns in this patient population.

## 2. Materials and Methods

### 2.1. Study Design and Patient Selection

This retrospective cohort study was conducted at a single tertiary referral center and reviewed consecutive elderly patients who underwent long thoracolumbar fusion terminating at L5 between January 2010 and December 2020. Long fusion was defined as fixation involving five or more vertebral levels, with the uppermost instrumented vertebra located at or above L1 and L5 as the lowest instrumented vertebra.

Inclusion criteria were as follows: (1) age ≥60 years at the time of surgery; (2) minimum follow-up duration of 24 months; (3) fusion length of ≥5 levels terminating at L5; (4) circumferential fusion performed at L4–L5; and (5) absence of a significant fractional lumbosacral curve in patients with degenerative lumbar scoliosis. Exclusion criteria included (1) previous decompression or fusion at L5–S1; (2) sacralization of L5; (3) spontaneous fusion of the L5–S1 segment caused by advanced osteophyte formation; (4) neuromuscular, congenital, infectious, traumatic, or neoplastic spinal disorders; and (5) incomplete radiographic or clinical follow-up.

**Figure 1:**
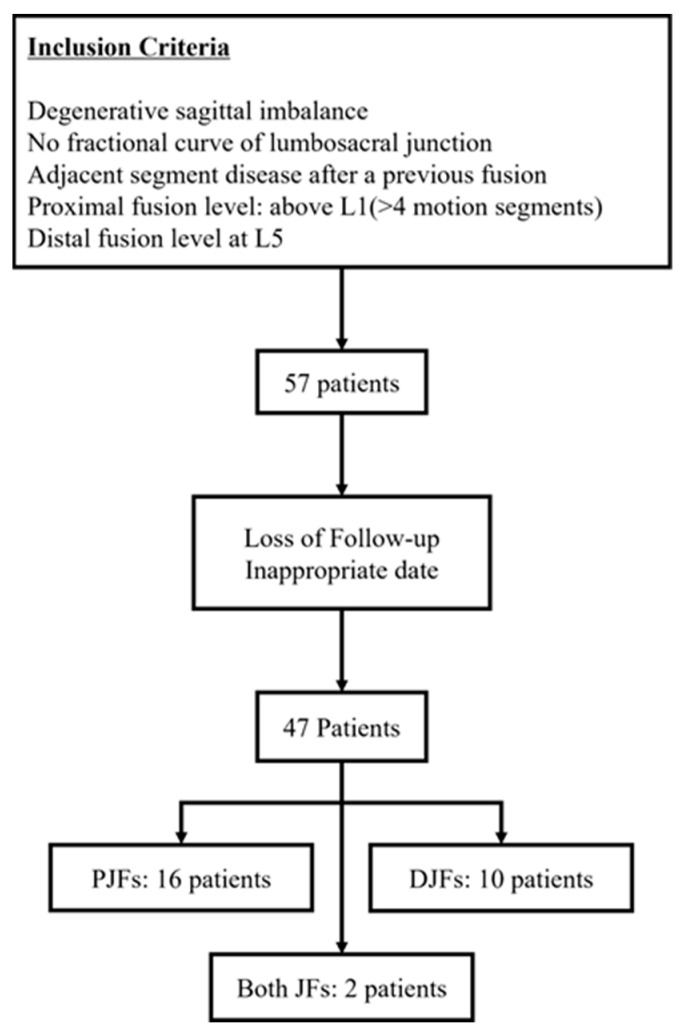
Flow diagram of patient selection.

Among 57 initially screened patients, 47 met all inclusion criteria and were included in the final analysis (Figure 1). Indications for surgery included degenerative lumbar scoliosis, degenerative sagittal imbalance, multilevel spinal stenosis, or revision surgery for adjacent segment degeneration with sagittal imbalance.

### 2.2. Surgical Procedure

All surgeries were performed via a posterior approach with segmental pedicle screw instrumentation. Long-segment fusion terminating at L5 was performed according to the extent of deformity and clinical symptoms.

Anterior column support at the L4–L5 level was selectively employed when additional segmental stability or sagittal correction was required, and circumferential fusion at L4–L5 was routinely performed to enhance distal fixation strength.

Sagittal alignment correction was achieved using posterior column osteotomies and rod contouring as needed, based on individual deformity characteristics. Sagittal correction strategies were individualized rather than standardized, considering patient age, baseline sagittal alignment, and overall clinical condition, rather than applying a uniform alignment target across all patients.

### 2.3. Radiographic Evaluation

Standing full-length anteroposterior and lateral radiographs were obtained preoperatively, within one month postoperatively, and at the final follow-up. Radiographic parameters included pelvic incidence (PI), pelvic tilt (PT), sacral slope (SS), lumbar lordosis (LL), thoracolumbar kyphosis (TLK), thoracic kyphosis (TK), C7 sagittal vertical axis (SVA), T1 pelvic angle (T1PA), proximal junctional angle (PJA), and distal junctional angle (DJA). DJA was defined as the angle between the inferior endplate of L5 and the superior endplate of S1.

All radiographic measurements were independently performed by two fellowship-trained spine surgeons who were blinded to clinical outcomes. Interobserver discrepancies were resolved through joint review, and consensus values were used for analysis

### 2.4. Assessment of L5–S1 Disc Degeneration

**Figure 2:**
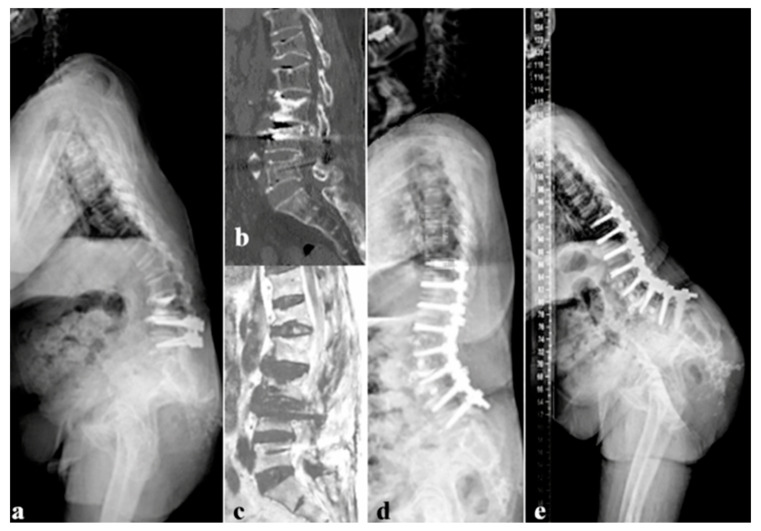
Representative case of distal junctional failure after long fusion to L5. (**a**) Preoperative standing lateral radiograph of a 69-year-old female patient demonstrating degenerative sagittal imbalance after vertebroplasty. SVA = 166.3 mm; PI = 54.5°; TK = −22.1°; LL = 21.5°; PT = 32.4°; SS = 21.9°; PI–LL = 76.0°; DJA = 12.4°; PJA = −15.5°. The modified Weiner grade for the L5–S1 disc was grade 0. (**b**) Preoperative computed tomography showing preserved L5–S1 disc height consistent with modified Weiner grade 0. (**c**) Preoperative magnetic resonance imaging demonstrating Pfirrmann grade C degeneration at L5–S1. (**d**) Postoperative standing lateral radiograph obtained four weeks after surgery demonstrating improved sagittal alignment. SVA = 60.4 mm; PI = 54.7°; TK = 21.7°; LL = 45.0°; PT = 21.3°; SS = 33.2°; PI-LL = 12.7°, DJA = 5.8° and PJA = 1.3°, however, LIV fracture developed at 3 months postoperatively. (**e**) Follow-up radiograph obtained four years after the index surgery demonstrating recurrent sagittal imbalance secondary to distal junctional failure.

Preoperative L5–S1 disc degeneration was evaluated using lateral radiographs according to the modified Weiner classification and magnetic resonance imaging (MRI) using the Pfirrmann grading system [11,12]. In cases of discordance between radiographic and MRI-based assessments, computed tomography was additionally reviewed. Final grading was determined by consensus among all authors. A representative case of distal junctional failure is shown in Figure 2.

### 2.5. Definition and Classification of Junctional Failure (Revised)

Proximal junctional kyphosis (PJK) was defined radiographically as a proximal junctional angle (PJA) greater than 10° and at least 10° greater than the preoperative measurement [15]. Proximal junctional failure (PJF) was diagnosed when PJK was accompanied by clinical symptoms and structural failure, including fracture of the upper instrumented vertebra (UIV) or UIV+1, junctional subluxation, implant loosening or breakage at the proximal junction, or the development of new neurological deficits requiring medical or surgical intervention [15,16].

Distal junctional kyphosis (DJK) was defined as a postoperative distal junctional angle (DJA) greater than 10° or an increase of more than 10° compared with the immediate postoperative measurement [17]. Distal junctional failure (DJF) was defined as symptomatic DJK associated with structural failure at the distal junction, including fracture of the L5 vertebra (lowest instrumented vertebra), loosening or pull-out of distal pedicle screws, rod or screw breakage, or progressive distal instability requiring revision surgery [8,17].

The proximal junctional angle (PJA) was measured between the inferior endplate of the upper instrumented vertebra and the superior endplate of the vertebra two levels above. The distal junctional angle (DJA) was measured between the inferior endplate of L5 and the superior endplate of S1.

Patients were classified into three groups according to the type of junctional failure observed during follow-up: no junctional failure, PJF, and DJF. Patients who developed both PJF and DJF were analyzed separately when appropriate.

### 2.6. Statistical Analysis

Statistical analyses were performed using IBM SPSS Statistics version 21.0 (IBM Corp., Armonk, NY, USA). Continuous variables were expressed as mean ± standard deviation and compared using the Kruskal–Wallis test, with post hoc pairwise comparisons performed using the Mann–Whitney U test with Bonferroni correction. Categorical variables were analyzed using Fisher’s exact test or the linear-by-linear association test, as appropriate.

Junctional failure–free survival was analyzed using the Kaplan–Meier method, and differences between groups were assessed using the log-rank test. Variables with a *p*-value < 0.10 in univariate analysis were entered into a multivariate Cox proportional hazards regression model to explore independent risk factors for PJF and DJF. Given the retrospective design and limited number of junctional failure events, multivariate Cox regression analyses were performed in an exploratory manner, and the results were interpreted with caution. A two-sided *p*-value < 0.05 was considered statistically significant.

## 3. Results

### 3.1. Patient Demographics and Surgical Characteristics

A total of 47 patients met the inclusion criteria and were included in the analysis. The mean age was 70.6 ± 5.7 years, and 37 patients (78.7%) were female. The mean body mass index was 24.1 ± 3.5 kg/m^2^, and the mean follow-up duration was 48.7 ± 43.9 months. The average number of fused levels was 6.2 ± 1.4 (range, 5–10).

Among the cohort, 28 patients (59.6%) developed junctional failure during follow-up, whereas 19 patients (40.4%) did not. Proximal junctional failure (PJF) occurred in 16 patients (34.0%), distal junctional failure (DJF) in 10 patients (21.3%), and both PJF and DJF in 2 patients (4.3%). There were no significant differences among the normal, PJF, and DJF groups in terms of age, sex distribution, body mass index, bone mineral density, or fusion length (Table 1).

### 3.2. Association Between Junctional Failure and Revision Surgery

Twenty-seven patients (57.4%) underwent primary index surgery, whereas 20 patients (42.6%) had a history of prior floating lumbar fusion. Junctional failure developed in 15 patients (55.6%) after primary surgery and in 12 patients (60.0%) following revision surgery.

Although distal junctional failure was more frequently observed in the revision surgery group than in the primary surgery group (35.0% vs. 18.5%), the overall distribution of junctional failure types did not differ significantly according to revision status (*p* = 0.300, Table 2).

### 3.3. Relationship Between Junctional Failure and L5–S1 Disc Degeneration

Preoperative L5–S1 disc degeneration, assessed using the modified Weiner classification, showed a statistically significant difference in distribution among junctional failure patterns (*p* = 0.024, Table 2). Among patients with minimal disc degeneration (Weiner grades 0–1), proximal junctional failure was more prevalent than distal junctional failure. In contrast, patients with moderate degeneration (grade ≥ 2) more frequently developed distal junctional failure.

Similarly, Pfirrmann grading based on magnetic resonance imaging demonstrated a significant difference in degeneration severity among the groups (*p* = 0.044). Higher Pfirrmann grades were more commonly observed in patients who developed distal junctional failure compared with those without junctional failure or with proximal failure.

### 3.4. Comparison of Spinopelvic Parameters Among Groups

Preoperative spinopelvic parameters were largely comparable among the three groups, except for thoracolumbar kyphosis (TLK) and proximal junctional angle (PJA), which differed significantly between the PJF and DJF groups. Preoperative T1 pelvic angle was significantly greater in the PJF group compared with patients without junctional failure.

At one month postoperatively, thoracic kyphosis and proximal junctional angle were significantly greater in the PJF group than in the DJF group. At the final follow-up, patients with junctional failure demonstrated significantly greater sagittal vertical axis and T1 pelvic angle values compared with patients without junctional failure. Detailed comparisons of radiographic parameters are summarized in Table 3.

### 3.5. Junctional Failure–Free Survival and Risk Factor Analysis

**Figure 3:**
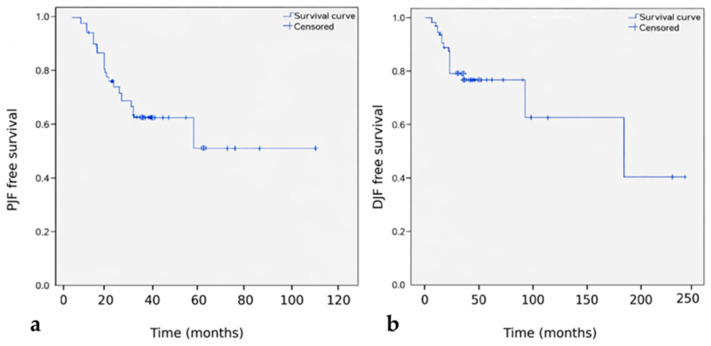
Junctional failure–free survival analysis. Kaplan–Meier curves demonstrating junctional failure–free survival for proximal junctional failure (PJF) (**a**) and distal junctional failure (DJF) (**b**) following long thoracolumbar fusion terminating at L5.

Kaplan–Meier survival analysis demonstrated that proximal junctional failure occurred earlier and more frequently than distal junctional failure (Figure 3). At one year postoperatively, the cumulative incidence of PJF and DJF was 14.9% and 8.5%, respectively. At two years, these values increased to 34.0% for PJF and 19.1% for DJF.

Multivariate Cox proportional hazards regression showed that preoperative thoracolumbar kyphosis, preoperative T1 pelvic angle, and postoperative pelvic incidence–lumbar lordosis mismatch were associated with proximal junctional failure. Postoperative proximal junctional angle and lumbar lordosis were associated with distal junctional failure (Table 4).

### 3.6. Reoperation Due to Junctional Failure

**Figure 4:**
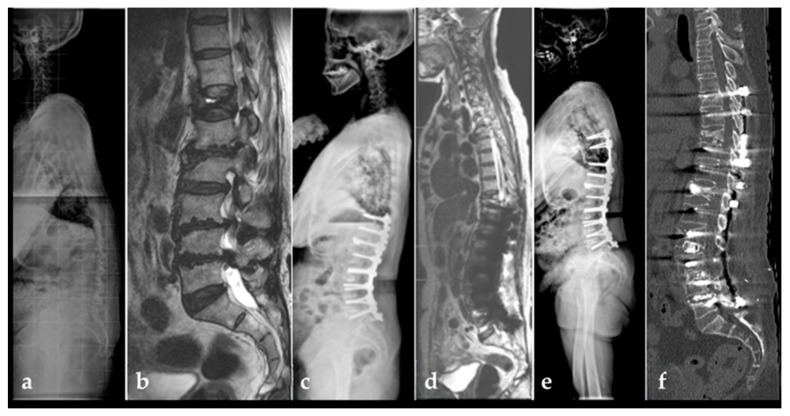
Representative case of proximal junctional failure. (**a**) Preoperative standing lateral radiograph of a 73-year-old female patient with degenerative lumbar scoliosis and sagittal imbalance, demonstrating an SVA = 105.2 mm; PI = 75.1°; TK = 13.1°; LL = −12.5°; PT = 47°; SS = 28.1°; PI-LL = 87.6°, DJA = 15°; and PJA = −4.0°. The modified Weiner grade for the L5–S1 disc was grade 0. (**b**) Preoperative magnetic resonance imaging demonstrating Pfirrmann grade C degeneration at L5–S1 with multilevel spinal stenosis. (**c**) Immediate postoperative standing lateral radiograph showing fusion from T10 to L5 with partial correction of sagittal alignment in SVA = 18.7 mm; PI = 75.6°; TK,14°; LL = −48.3°; PT, 41.2°; SS, 36.6°; PI-LL, 27.3°, DJA,15° and PJA −4.0°, indicating residual undercorrection. (**d**) Magnetic resonance imaging obtained four months postoperatively demonstrating a fracture at the upper instrumented vertebra plus one level (UIV+1), consistent with proximal junctional failure. (**e**) Follow-up standing lateral radiograph obtained two years after revision surgery demonstrating restoration of sagittal alignment without progression of L5–S1 disc degeneration. (**f**) Computed tomography obtained 28 months after the index surgery confirming the absence of advanced degeneration at the L5–S1 segment.

Nine of the 28 patients with junctional failure (32.1%) required reoperation, representing 19.1% of the entire cohort. Reoperation was more commonly performed for proximal junctional failure than for distal junctional failure. No patient underwent revision surgery solely for distal junctional failure without concomitant proximal pathology (Figure 4).

## 4. Discussion

The present study evaluated risk factors for junctional failure following long thoracolumbar fusion terminating at L5, with particular emphasis on the relative contributions of residual L5–S1 disc degeneration and sagittal spinopelvic alignment. The principal finding is that sagittal alignment parameters appear to play a more prominent role in both the occurrence and pattern of junctional failure than preoperative disc degeneration at L5–S1. Furthermore, proximal and distal junctional failures demonstrated distinct radiographic and biomechanical profiles, supporting the concept that these entities represent different modes of postoperative mechanical failure rather than a single continuous spectrum [1,3,4,5,6,7].

The decision to terminate a long fusion at L5 or extend fixation to the sacrum remains one of the most debated issues in adult spinal deformity surgery, particularly in elderly patients [1,3,4]. Fusion stopping at L5 preserves lumbosacral motion and may reduce operative time, blood loss, and perioperative morbidity; however, it also places substantial mechanical demands on the remaining L5–S1 segment. Previous studies have reported variable rates of postoperative sagittal decompensation and reoperation following long fusion to L5, underscoring the need to better define patient- and alignment-specific risk factors [1,2,4,9]. From a clinical decision-making perspective, our findings suggest that elderly patients with pronounced sagittal imbalance, such as elevated preoperative T1 pelvic angle or residual postoperative PI–LL mismatch, may be suboptimal candidates for fusion terminating at L5. In such cases, extension of fixation to the sacrum or ilium should be considered to enhance mechanical stability and reduce the risk of junctional failure.

In the current cohort, preoperative L5–S1 disc degeneration was significantly associated with the pattern of junctional failure. Patients with minimal degeneration (modified Weiner grades 0–1) more frequently developed proximal junctional failure, whereas those with more advanced degeneration (grade ≥ 2) were more likely to experience distal junctional failure. Similar trends have been reported by Witiw et al. [4], who demonstrated that preserved L5–S1 disc integrity was associated with a higher incidence of proximal junctional pathology, whereas advanced degeneration increased the risk of distal failure. These findings may suggest that a relatively mobile lumbosacral segment may contribute to increased stress transfer to the proximal junction following long fusion, whereas structural compromise at L5–S1 predisposes patients to distal mechanical breakdown. From a long-term perspective, these findings highlight a trade-off between preserving L5–S1 motion and ensuring mechanical stability in long-segment fusion. While motion preservation may be advantageous in selected patients, a relatively mobile lumbosacral junction can increase stress transfer to the proximal junction in certain sagittal alignment contexts; therefore, the decision to preserve or sacrifice L5–S1 should be individualized.

Despite this association, disc degeneration alone did not fully account for junctional failure occurrence. In our study, sagittal spinopelvic alignment parameters were more consistently associated with mechanical failure than disc degeneration. Preoperative thoracolumbar kyphosis and T1 pelvic angle, as well as postoperative pelvic incidence–lumbar lordosis (PI–LL) mismatch, were independently associated with proximal junctional failure. These findings are consistent with prior studies demonstrating that insufficient restoration of sagittal alignment increases proximal stress concentration and predisposes patients to junctional collapse [10,16].

Distal junctional failure demonstrated a different risk profile. Postoperative proximal junctional angle and lumbar lordosis were independently associated with DJF, suggesting that distal failure is closely related to residual sagittal imbalance and local junctional mechanics rather than preoperative disc status alone. Previous biomechanical and clinical studies have shown that inadequate lumbar lordosis correction, particularly in patients with high pelvic incidence, results in increased shear and bending moments at the distal end of long constructs [14,18,19]. Our findings support these observations and highlight the importance of achieving appropriate global and segmental alignment when selecting L5 as the distal fusion level. Although specific age-adjusted alignment thresholds could not be established in the present study, these findings support the concept that sagittal correction goals in elderly patients should be individualized according to patient age and baseline alignment rather than applying a uniform target. Future studies are warranted to determine whether age-adjusted parameters, such as PI–LL mismatch or T1 pelvic angle thresholds, may further reduce the risk of junctional failure.

The angle thresholds used to define proximal and distal junctional kyphosis in this study were based on widely accepted and validated criteria. Proximal junctional kyphosis was defined as a proximal junctional angle exceeding 10° and increasing by more than 10° relative to the preoperative value, as originally described by Glattes et al. [15]. Distal junctional kyphosis was defined using similar angular criteria, consistent with definitions proposed in previous studies evaluating distal junctional pathology and mechanical failure [17,18]. These thresholds have been adopted in multiple large series and systematic reviews, providing a reproducible framework for distinguishing clinically meaningful junctional deformity from expected postoperative alignment changes [8,17].

Measurement reliability is a critical consideration when evaluating sagittal alignment and junctional angles. In the present study, all radiographic parameters were independently assessed by two fellowship-trained spine surgeons who were blinded to clinical outcomes. Interobserver agreement was high, with intraclass correlation coefficients exceeding 0.80 for all key sagittal alignment parameters. This level of reliability is comparable to that reported in previous alignment-focused studies and minimizes the likelihood that observer variability influenced the observed associations [15].

Interestingly, although a deep-seated L5 vertebra has been biomechanically implicated as a potential contributor to increased stress concentration at the lumbosacral junction, no significant association was observed between intercristal line position and junctional failure in this cohort. This finding suggests that global sagittal alignment may outweigh local anatomical variations in determining postoperative mechanical outcomes. Similar conclusions have been reported in studies emphasizing alignment-based surgical planning over isolated anatomical factors [10,13,14].

Several limitations of this study should be acknowledged. First, the retrospective design and relatively small sample size, particularly after subdivision into multiple junctional failure patterns, may limit statistical power and increase the risk of type I error and overfitting in multivariable Cox regression analyses. Second, the absence of a control group undergoing fusion to the sacrum precludes direct comparison between distal fusion strategies and limits determination of whether the observed junctional failure patterns are specific to fusion terminating at L5 or reflect a general consequence of long spinal fusion in elderly patients. Third, assessment of L5–S1 disc degeneration relied on integration of radiographic, MRI, and selectively CT-based evaluations with final author consensus, without formal quantitative assessment of inter-method reliability, and disc degeneration was treated as a static variable without evaluation of its temporal progression. Fourth, interpretation of sagittal alignment parameters may be limited by modest statistical significance in some intergroup comparisons, lack of correction for multiple comparisons, and potential multicollinearity among biomechanically related variables. Lastly, potentially confounding factors such as bone quality, construct stiffness, osteotomy techniques, and proximal reinforcement strategies were not systematically analyzed, and although the mean follow-up duration exceeded four years, longer-term follow-up may be required to fully capture late-onset junctional failures. Accordingly, the present findings should be interpreted as exploratory and hypothesis-generating rather than definitive evidence.

## 5. Conclusions

Sagittal spinopelvic alignment parameters appear to play a more prominent role in junctional failure patterns than residual L5–S1 disc degeneration in long spinal fusions ending at L5. Proximal and distal junctional failures represent distinct mechanical entities with different radiographic and biomechanical risk profiles. Careful preoperative assessment and adequate restoration of sagittal alignment should be prioritized when selecting L5 as the distal fusion level.

## Figures and Tables

**Table 1:** Comparison of demographic and baseline characteristics among groups.

	Normal (*n* = 19)	PJF (*n* = 16)	DJF (*n* = 10)	*p*-Value
Gender (F:M)	13:6	16:0	7:3	0.569
Age (years)	70.1 ± 5.5	70.6 ± 6.3	71.2 ± 5.8	0.874
BMI (kg/m^2^)	24.2 ± 3.2	23.8 ± 4.4	24.2 ± 2.6	0.794
BMD (T-score)	−1.24 ± 1.14	−1.03 ± 2.15	−1.11 ± 1.25	0.625
Fusion length (*n*)	5.3 ± 1.5	5.4 ± 1.6	5.2 ± 1.4	0.502
Follow-up period (months)	39.4 ± 22.5	65.1 ± 67.9	44.3 ± 19.7	0.275
Intercristal line (0:1:2)	9:0:10	10:0:6	6:1:3	0.314
Weiner grade of L5-S1 (0:1:2:3)	15:2:2:0	10:4:1:1	4:1:5:0	0.024 *
Pfirrmann grade of L5-S1 (2:3:4)	1:17:1	2:13:1	0:7:3	0.124
Reoperation due to JF (*n*, %)	-	6 (37.5%)	2 (20%)	0.420

BMI = Body Mass Index; BMD = Bone Mineral Density; PJF = Proximal Junctional Failure; DJF = Distal Junctional Failure; JF = junctional failure. Values are shown as numbers (%) or mean ± SD unless indicated otherwise. * Significant difference.

**Table 2:** Comparison of occurrence of junctional failure types according to each factor.

	Normal (*n* = 19)	PJF (*n* = 16)	DJF (*n* = 10)	Both JF (*n* = 2)	*p*-Value
Revision surgery					0.300
No (*n*)	12	10	4	1	
Yes (*n*)	7	5	6	1	
Weiner grade					0.024 *
0 (*n*)	15	10	4	1	
1 (*n*)	2	4	1	0	
2 (*n*)	2	1	5	1	
3 (*n*)	0	1	0	0	
Pfirrmann grade					0.044 *
2 (*n*)	1	2	0	0	
3 (*n*)	17	13	7	1	
4 (*n*)	1	1	3	1	
Sagittal balance					0.403
Normal (*n*)	5	5	2	0	
Positive (*n*)	6	6	7	2	
Negative (*n*)	8	5	1	0	

PJF = Proximal Junctional Failure; DJF = Distal Junctional Failure; JF = junctional failure. * Significant difference.

**Table 3:** Comparison of Spinopelvic Parameters Among Normal, PJF, and DJF Groups.

	Normal (*n* = 19)	PJF (*n* = 16)	DJF (*n* = 10)	*p*-Value
Preoperative				
PI (°)	52.3 ± 8.9	58.0 ± 11.8	54.9 ± 13.5	NS
PT (°)	22.7 ± 9.3	30.6 ± 10.6	24.9 ± 8.4	NS
SS (°)	29.4 ± 9.0	27.9 ± 10.8	29.6 ± 8.9	NS
LL (°)	31.4 ± 18.3	20.1 ± 16.1	21.9 ± 23.9	NS
PI-LL (°)	20.9 ± 19.9	37.9 ± 18.7	33.1 ± 20.4	NS
TLK (°)	10.8 ± 11.1	21.7 ± 19.1	1.2 ± 16.6	PJF vs. DJF: 0.010
TK (°)	16.4 ± 14.1	16.4 ± 14.1	17.2 ± 9.2	NS
SVA (cm)	5.5 ± 5.2	11.6 ± 7.9	10.4 ± 8.3	NS
T1PA (°)	22.4 ± 11.7	35.2 ± 12.8	28.4 ± 13.1	Normal vs. PJF: 0.010
PJA (°)	5.6 ± 10.6	9.1 ± 11.8	−2.1 ± 10.1	PJF vs. DJF: 0.014
DJA (°)	−13.0 ± 8.0	−10.5 ± 11.0	−14.2 ± 14.7	NS
Postoperative				
PT (°)	22.8 ± 16.3	26.8 ± 11.1	23.7 ± 9.4	NS
SS (°)	33.2 ± 7.8	29.9 ± 8.4	31.2 ± 7.9	NS
LL (°)	53.0 ± 10.6	45.9 ± 17.3	33.2 ± 15.9	NS
PI-LL (°)	−1.9 ± 14.4	10.8 ± 22.8	21.0 ± 14.8	NS
TLK (°)	10.5 ± 8.7	14.4 ± 11.9	2.1 ± 13.8	NS
TK (°)	28.5 ± 11.6	31.7 ± 13.2	17.0 ± 10.3	PJF vs. DJF: 0.009
SVA (cm)	1.8 ± 4.6	4.4 ± 7.4	7.6 ± 5.6	NS
T1PA (°)	14.7 ± 8.9	25.2 ± 13.9	24.6 ± 10.3	NS
PJA (°)	13.5 ± 6.4	16.8 ± 9.2	3.3 ± 9.5	PJF vs. DJF: 0.001
DJA (°)	−10.9 ± 7.1	−10.0 ± 5.6	−7.6 ± 11.2	NS
Last follow-up				
PT (°)	21.7 ± 12.2	28.4 ± 8.9	25.6 ± 9.2	NS
SS (°)	30.9 ± 7.2	28.8 ± 11.4	30.1 ± 6.9	NS
LL (°)	51.3 ± 11.1	44.3 ± 18.8	28.4 ± 16.7	NS
PI-LL (°)	0.4 ± 14.5	12.6 ± 23.7	27.6 ± 16.2	NS
TLK (°)	12.0 ± 9.9	14.3 ± 14.1	1.9 ± 7.3	NS
TK (°)	29.3 ± 11.7	29.9 ± 23.2	17.8 ± 9.7	PJF vs. DJF: 0.004
SVA (cm)	1.7 ± 4.7	8.7 ± 8.1	11.2 ± 7.1	Normal vs. PJF: 0.008 Normal vs. DJF: 0.001
T1PA (°)	14.4 ± 9.4	28.1 ± 14.7	30.0 ± 13.1	Normal vs. PJF: 0.008 Normal vs. DJF: 0.001
PJA (°)	11.8 ± 7.4	21.5 ± 11.8	3.9 ± 8.6	Normal vs. PJF: 0.005 PJF vs. DJF: <0.001
DJA (°)	−9.5 ± 9.0	−9.5 ± 6.9	−2.5 ± 9.8	NS

*p*-values indicate comparisons between groups. NS = not significant. Only statistically significant values (*p* < 0.05) are indicated.

**Table 4:** Risk factor analysis for each PJF and DJF.

	Univariate Analysis	Multivariate Analysis		
	*p*-value	HR	95% CI	*p*-value
PJF				
Gender	0.065	0.045	0.002–0.915	0.044 *
Postoperative PI-LL	0.050	0.877	0.777–0.989	0.033 *
Preoperative TLK	0.010 *	1.164	1.058–1.280	0.002 *
Preoperative TPA	0.016 *	1.269	1.030–1.565	0.025 *
DJF				
Postoperative PJA	0.014 *	0.899	0.822–0.983	0.019 *
Postoperative LL	0.001 *	0.920	0.863–0.981	0.011 *

* Significant difference.

## Data Availability

The datasets used and analyzed during the current study are available from the corresponding author on reasonable request.

## Abbreviations

The following abbreviations are used in this manuscript:

ASD	Adult spinal deformity
DJF	Distal junctional failure
LL	Lumbar lordosis
MRI	Magnetic resonance imaging
PI	Pelvic incidence
PT	Pelvic tilt
PJF	Proximal junctional failure
PSF	Posterior spinal fusion
SVA	Sagittal vertical axis
